# A novel finite-time average consensus protocol based on event-triggered nonlinear control strategy for multiagent systems

**DOI:** 10.1186/s13660-017-1533-6

**Published:** 2017-10-16

**Authors:** Xiaobo Wang, Juelong Li, Jianchun Xing, Ronghao Wang

**Affiliations:** 1 College of Defense Engineering, PLA University of Science and Technology, Nanjing, 210007 China; 2 Research Center of Costal Defense Engineering, Beijing, 100841 China

**Keywords:** multiagent system, finite-time, event-triggered control, consensus, protocol

## Abstract

We present a novel finite-time average consensus protocol based on event-triggered control strategy for multiagent systems. The system stability is proved. The lower bound of the interevent time is obtained to guarantee that there is no Zeno behavior. Moreover, the upper bound of the convergence time is obtained. The relationship between the convergence time and protocol parameter with initial state is analyzed. Lastly, simulations are conducted to verify the effectiveness of the results.

## Introduction

In recent years, many applications required a lot of vehicles or robots to work cooperatively and accomplish a complicated task. Given this, many researchers have devoted themselves to the studies of coordination control of multiagent systems [1, 2]. The primary researches in this field include the problems of consensus [3], flocking [4], formation control [5, 6], collective behavior of swarms [7, 8], etc. Of these, the issue of consensus is the basis of studying other problems. Multiagent consensus refers to the design of a proper consensus protocol based on the local information of each agent such that all agents can reach an agreement with regard to certain quantities of interest [4].

In practical multiagent systems, each agent is usually equipped with a small embedded microprocessor and has limited energy, which usually has only limited computing power and working time. These disadvantages drive researchers to develop event-triggered control schemes, and some important achievements have been made recently [9–11]. For example, in [12] the authors introduced the deterministic event-triggered strategy to develop consensus control algorithms, and the lower bound of the interevent time was obtained to guarantee that there is no Zeno behavior. In [13] the problems of event-triggered integrated fault detection, isolation, and control for discrete-time linear systems were considered. It was shown that the amount of data that is sent through the sensor-to-filter and filter-to-actuator channels are dramatically decreased by using an event-triggered technique applied to both the sensor and filter nodes. In [14], the authors proposed a new multiagent consensus event-based control approach. The measurement broadcasts were scheduled in an event-based fashion, and the continuous monitoring of each neighbor’s state is no longer demanded. In [15] the authors proposed a combinational measurement strategy to event design and developed a new event-triggered control algorithm. In this strategy, each agent is only triggered at his own event time, which lowers the frequency of controller updates and reduces the amount of communication. In [16] the authors proposed a self-triggered consensus algorithm for multiagent systems. The algorithm is simpler in formulation and computation. Thus, more energy can be saved using the proposed algorithm in practical multiagent systems.

Moreover, the convergence time is a significant performance indicator for a consensus protocol in the study of the consensus problem. In most works the protocols only achieve state consensus in infinite time interval, that is, the consensus is only achieved asymptotically. However, the stability or performance of multiagent systems in a finite time interval needs to be considered in several cases. The finite-time stability focuses on the behavior of system responses over a finite time interval [17, 18]. Therefore, studying the finite-time stability of multiagent systems is valuable to some degree. The multiagent finite-time stability analysis has also elicited the attention of many researchers [19–22].

Recently, there are few results reported in the literature to address finite-time event-triggered control consensus protocols for multiagent systems. To the best of our knowledge, in [23] the authors presented two novel nonlinear consensus protocols based on event-driven strategy to investigate the finite-time consensus problem of leaderless and leader-following multiagent systems. However, many parameters exist in the proposed protocols, making the protocols complex and restricted, and the relationship between the convergence time and parameters is unclear. Inspired by these, a new consensus protocol based on the event-triggered control strategy is proposed in this paper.

The main contributions of this paper can be summarized as follows: (1) a new finite-time consensus protocol based on the event-triggered control strategy for multiagent systems is presented, and the system stability is proved. The protocol is simpler in formulation and computation. (2) The lower bound of the inter-event time is gotten to guarantee there is no Zeno behavior. (3) The upper bound of convergence time is obtained. The relationship between the convergence time and protocol parameter, the initial state, is analyzed.

The rest of this paper is organized as follows. In Section 2, we introduce some essential background and present the problem statement. The main results and the proof are provided in Section 3. The simulation results are shown in Section 4, and the conclusions and the future works are provided in Section 5.

### Notations

$\mathbf{1}= [1,1,\ldots,1]^{\mathrm{T}}$ with compatible dimensions, $\mathcal{I}_{n} = \{ 1,2,\ldots,n\}$. $|x|$ is the absolute value of a real number *x*, $\|\cdot\|$ denotes the 2-norm in $R^{n}$, $\operatorname{span}\{ \mathbf{1}\} = \{\boldsymbol{\varepsilon} \in R^{n}:\boldsymbol{\varepsilon} = r\mathbf{1},r \in R\}$, and *E* is the Euler number (approximately 2.71828).

## Background and problem statement

### Preliminaries

In this subsection, we introduce some basic definitions and results of algebraic graph theory. Comprehensive conclusions on algebraic graph theory are found in [24]. Moreover, we present two essential lemmas.

For an undirected graph $\mathcal{G} = ( \mathcal {V},\mathcal{E},\mathcal{A} )$ with *n* vertices, $\mathcal{V} = \{ v_{1},v_{2},\ldots,v_{n}\}$ is the vertex set, and $\mathcal{E}$ is the edge set, the adjacency matrix $\mathcal{A} = [a_{ij}]$ is the $n \times n$ matrix defined by $a _{ij}=1$ for $(i,j) \in\mathcal{E}$ and $a _{ij}=0$ otherwise. The neighbors of a vertex $v _{i}$ are denoted by $\mathcal {N}_{i} = \{ v_{j} \in\mathcal{V}:(v_{i},v_{j}) \in \mathcal{E}\}$, and then the vertices $v _{i}$ and $v _{j}$ are called adjacent. A path from $v _{i}$ to $v _{j}$ is a sequence of distinct vertices starting with $v _{i}$ and ending with $v _{j}$ such that consecutive vertices are adjacent. A graph is regarded as connected if there is a path between any two distinct vertices. The Laplacian of a graph $L= [l _{ij}] \in R^{n \times n}$ is defined by $l_{ij} = \sum_{k = 1,k \ne i}^{n} a_{ik}$ for $i=j$ and $l _{ij}=- a _{ij}$ otherwise. *L* has always a zero eigenvalue, and **1** is the associated eigenvector. We denote the eigenvalues of *L* by $0= \lambda _{1}(L)\leq\lambda_{2}(L)\leq\cdots\leq\lambda _{n}(L)$. For an undirected and connected graph, $\lambda_{2}(L) = \min_{\boldsymbol{\varepsilon} \ne0,1^{T}\boldsymbol{\varepsilon} = 0}\frac{\boldsymbol{\varepsilon}^{\mathrm{T}}L\boldsymbol{\varepsilon}}{ \boldsymbol{\varepsilon}^{\mathrm{T}}\boldsymbol{\varepsilon}} > 0$. Therefore, if $\mathbf{1}^{\mathrm{T}} \boldsymbol{\varepsilon} = 0$, then $\boldsymbol{\varepsilon} ^{\mathrm{T}} L \boldsymbol{\varepsilon} \geq \lambda_{2}(L) \boldsymbol{\varepsilon} ^{\mathrm{T}} \boldsymbol{\varepsilon} $.

#### Lemma 1

([25])

*Suppose that a function* $V(t): [0, \infty) \rightarrow[0, \infty)$ *is differentiable and satisfies the condition*

1 $$ \frac{dV(t)}{dt} \le- KV(t)^{\alpha}, $$

*where* $K >0$ *and* $0 < \alpha <1$. *Then* $V(t)$ *reaches zero at* $t ^{*}$, *and* $V(t) =0$ *for all* $t \geq t ^{*}$, *where*

2 $$ t^{*} = \frac{V(0)^{1 - \alpha}}{K(1 - \alpha)}. $$

#### Lemma 2

([26])

*If* $\xi_{1},\xi_{2}, \ldots,\xi_{n} \ge 0$ *and* $0< p \leq1$, *then*

3 $$ \Biggl( \sum_{i = 1}^{n} \xi_{i} \Biggr)^{p} \le\sum_{i = 1}^{n} \xi_{i}^{p} \le n^{1 - p} \Biggl( \sum _{i = 1}^{n} \xi_{i} \Biggr)^{p}. $$

### Problem formulation

The multiagent system investigated in this study consists of *n* agents, and the state of agent *i* is denoted by $x _{i}$, $i \in\mathcal{I}_{n}$. Denote the vector $\mathbf{x} =[x _{1}, x _{2}, \ldots, x _{n}]^{\mathrm{T}}$. The dimension of $x _{i}$ can be arbitrary as long as it is the same for all agents. For simplicity, we only analyze the one-dimensional case. The results are still valid for multidimensional cases through the introduction of the Kronecker product and inequality (3). We suppose that each agent satisfies the dynamics

4 $$ \dot{x}_{i}(t) = u_{i}(t), $$

where $u _{i}$ is the protocol, which is designed based on the state information received by the corresponding agent from its neighbors.

With the given protocol $u _{i}$, for any initial state, if there is a stable equilibrium $x ^{*}$ and a time $t ^{*}$ satisfying $x_{i}= x ^{*}$ for all $i \in\mathcal{I}_{n}$ with $t \geq t ^{*}$, then the finite-time consensus problem is solved [25]. In addition, the average consensus problem is solved if the final consensus state is the average value of the initial state, namely, $x_{i}(t) = \sum_{i = 1}^{n} x_{i} (0) / n$ for all $i \in\mathcal{I}_{n}$ with $t \to\infty$.

### Event-triggered control consensus protocol

In the event design, we suppose that each agent can measure its own state $x _{i}(t)$ and obtain its neighbors’ states stably. For each vertex $v_{i}$ and $t \geq 0$, introduce a measurement error $e _{i}(t)$ and denote the vector $\mathbf{e} (t)=[e _{1}(t), e _{2}(t),\ldots,e _{n}(t)]$. The time instants at which the events are triggered are defined by the condition $f(e(t _{i}))=0$. Suppose the triggering time sequence of vertex $v _{i}$ is $t^{i} = 0,\tau_{1}, \ldots,\tau_{s}^{i}, \ldots$ .

Between events are triggered, the value of the input *u* is held constant and can be formalized as

5 $$ u_{i}(t) = u_{i}(\tau_{s}),\quad t \in [ \tau_{s},\tau_{s + 1} ). $$

It is well known that the control algorithm is a piecewise constant function, and the value of the input is equal to the last control update.

The protocol based on event-triggered control utilized to solve the finite-time average consensus problem is

6 $$ u_{i} ( t ) = \sum_{j = 1}^{n} a_{ij} \operatorname{sign} \bigl( x_{j}(\tau_{s}) - x_{i}(\tau_{s}) \bigr) \bigl\vert x_{j}( \tau_{s}) - x_{i}(\tau_{s}) \bigr\vert ^{\alpha},\quad t \in [ \tau_{s},\tau_{s + 1} ), $$

where $0< \alpha<1$, and the $\operatorname{sign}(\cdot)$ is the sign function defined as

7 $$ \operatorname{sign}( x) = \textstyle\begin{cases} 1,& x > 0,\\ 0,& x = 0,\\ - 1,& x < 0. \end{cases} $$

## Main results

### Stability analysis

In this subsection, we study protocol (6). Now, we are in a position to present our main results.

#### Theorem 1

*Suppose that the communication topology of a multiagent system is undirected and connected and that the triggered function is given by*

8 $$ f_{i} \bigl( t,e_{i}(t),\delta_{i}(t) \bigr) = \bigl\Vert e_{i}(t) \bigr\Vert - \biggl( \frac{\mu ( 4\lambda_{2} ( D ) )^{ ( 1 + \alpha ) / 2}}{2^{ ( 1 - \alpha ) / 2}} \biggr)^{1 / \alpha} \bigl\Vert \delta_{i}(t) \bigr\Vert , $$

*where* $D = [d_{ij}] \in R^{n \times n}$, $d_{ij} = (a_{ij})^{2 / ( 1 + \alpha )}$, *and* $0< \mu<1$.

*Then*, *protocol* (6) *solves the finite*-*time average consensus problem for any initial state*. *Moreover*, *the settling time* *T* *satisfies*

9 $$ T \le\frac{4V(0)^{ ( 1 - \alpha ) / 2}}{ ( 1 - \mu ) ( 4\lambda_{2} ( D ) )^{ ( 1 + \alpha ) / 2}(1 - \alpha)}. $$

#### Proof

Given that the topology is undirected and connected, $a _{ij} = a _{ji}$ for all $i,j \in\mathcal{I}_{n}$. We obtain

10 $$ \sum_{i = 1}^{n} \dot{x}_{i}(t) = 0. $$

Let

11 $$ \kappa= \frac{1}{n}\sum_{i = 1}^{n} x_{i}(t). $$

Therefore, *κ* is time invariant.

Consequently,

12 $$ \mathbf{1}^{\mathrm{T}}\boldsymbol{\delta} (t) = \sum _{i = 1}^{n} x_{i} (t) - n\kappa= \sum _{i = 1}^{n} x_{i} (t) - \sum _{i = 1}^{n} x_{i} (t) = 0. $$

Define the measurement error as follows:

13 $$\begin{aligned} e_{i}(t) =& \Biggl( \sum_{j = 1}^{n} a_{ij} \operatorname{sign} \bigl( x_{j}(\tau_{s}) - x_{i}(\tau_{s}) \bigr) \bigl( x_{j}( \tau_{s}) - x_{i}(\tau_{s}) \bigr)^{\alpha} \\ &{}- \sum_{j = 1}^{n} a_{ij} \operatorname{sign} \bigl( x_{j}(t) - x_{i}(t) \bigr) \bigl\vert x_{j}(t) - x_{i}(t) \bigr\vert ^{\alpha} \Biggr)^{1 / \alpha},\quad t \in [ \tau_{s},\tau_{s + 1} ). \end{aligned}$$

Then we get

14 $$\begin{aligned} e_{i}^{\alpha} (t) =& \sum_{j = 1}^{n} a_{ij} \operatorname{sign} \bigl( x_{j}(\tau_{s}) - x_{i}(\tau_{s}) \bigr) \bigl( x_{j}( \tau_{s}) - x_{i}(\tau_{s}) \bigr)^{\alpha} \\ &{}- \sum_{j = 1}^{n} a_{ij} \operatorname{sign} \bigl( x_{j}(t) - x_{i}(t) \bigr) \bigl\vert x_{j}(t) - x_{i}(t) \bigr\vert ^{\alpha}. \end{aligned}$$

Let $\boldsymbol{\delta} (t) = [\delta_{1}(t),\delta_{2}(t), \ldots,\delta_{n}(t)]^{\mathrm{T}}$ and $x_{i}(t) = \kappa+ \delta_{i}(t)$. Thus,

15 $$ \dot{\delta}_{i}(t) = \dot{x}_{i}(t) = \sum _{j = 1}^{n} a_{ij} \operatorname{sign} \bigl( x_{j}(t) - x_{i}(t) \bigr) \bigl\vert x_{j}(t) - x_{i}(t) \bigr\vert ^{\alpha} + e_{i}^{\alpha} (t). $$

Here we have taken the Lyapunov function

16 $$ V(t) = \frac{1}{2}\delta^{\mathrm{T}}\delta. $$

By differentiating $V(t)$ we obtain

17 $$\begin{aligned} \frac{dV(t)}{dt} =& \sum_{i = 1}^{n} \delta_{i} \dot{\delta}_{i} \\ =& \sum_{i = 1}^{n} \delta_{i} \Biggl( \sum_{j = 1}^{n} a_{ij} \operatorname{sign} \bigl( x_{j}(t) - x_{i}(t) \bigr) \bigl\vert x_{j}(t) - x_{i}(t) \bigr\vert ^{\alpha} + e_{i}^{\alpha} (t) \Biggr) \\ =& \sum_{i = 1}^{n} \delta_{i} \Biggl( \sum_{j = 1}^{n} a_{ij} \operatorname{sign} \bigl( \delta_{j}(t) - \delta_{i}(t) \bigr) \bigl\vert \delta_{j}(t) - \delta_{i}(t) \bigr\vert ^{\alpha} + e_{i}^{\alpha} (t) \Biggr) \\ =& \sum_{i = 1}^{n} \delta_{i} \sum_{j = 1}^{n} a_{ij} \operatorname{sign} \bigl( \delta_{j}(t) - \delta_{i}(t) \bigr) \bigl\vert \delta_{j}(t) - \delta_{i}(t) \bigr\vert ^{\alpha} + \sum_{i = 1}^{n} \delta_{i} e_{i}^{\alpha} (t) \\ =& \frac{1}{2}\sum_{i,j = 1}^{n} a_{ij}\bigl( \delta_{i}\operatorname{sign} \bigl( \delta_{j}(t) - \delta_{i}(t) \bigr) \bigl\vert \delta_{j}(t) - \delta_{i}(t) \bigr\vert ^{\alpha} \\ &{} + \delta_{j}\operatorname{sign} \bigl( \delta_{i}(t) - \delta_{j}(t) \bigr) \bigl\vert \delta_{i}(t) - \delta_{j}(t) \bigr\vert ^{\alpha} \bigr) + \sum _{i = 1}^{n} \delta_{i} e_{i}^{\alpha} (t) \\ =& - \frac{1}{2}\sum_{i,j = 1}^{n} a_{ij} \bigl\vert \delta_{j}(t) - \delta_{i}(t) \bigr\vert ^{1 + \alpha} + \sum_{i = 1}^{n} \delta_{i} e_{i}^{\alpha} (t) \\ =& - \frac{1}{2}\sum_{i,j = 1}^{n} \bigl( (a_{ij})^{2 / ( 1 + \alpha )} \bigl( \delta_{j}(t) - \delta_{i}(t) \bigr)^{2} \bigr)^{ ( 1 + \alpha ) / 2} + \sum _{i = 1}^{n} \delta_{i} e_{i}^{\alpha} (t). \end{aligned}$$

Equation (17) results from $\delta_{j} - \delta_{i} = x_{j} - x_{i}$. For $0 < \alpha< 1$, we have $0.5 < (1+ \alpha)/2 < 1$. Denote

18 $$ \begin{aligned} &S_{1}(t) = - \frac{1}{2}\sum _{i,j = 1}^{n} \bigl( (a_{ij})^{2 / ( 1 + \alpha )} \bigl( \delta_{j}(t) - \delta_{i}(t) \bigr)^{2} \bigr)^{ ( 1 + \alpha ) / 2}, \\ &S_{2}(t) = \sum_{i = 1}^{n} \delta_{i} e_{i}^{\alpha} (t). \end{aligned} $$

Suppose that $V(t) \neq 0$; then $\boldsymbol{\delta} (t) \neq 0$. By Lemma 2 we have

19 $$\begin{aligned} S_{1}(t) \le&- \frac{1}{2} \Biggl( \sum _{i,j = 1}^{n} (a_{ij})^{2 / ( 1 + \alpha )} \bigl( \delta_{j}(t) - \delta_{i}(t) \bigr)^{2} \Biggr)^{ ( 1 + \alpha ) / 2} \\ =& - \frac{1}{2} \biggl( \frac{\sum_{i,j = 1}^{n} (a_{ij})^{2 / ( 1 + \alpha )} ( \delta_{j}(t) - \delta_{i}(t) )^{2}}{V(t)}V(t) \biggr)^{ ( 1 + \alpha ) / 2} \\ =& - \frac{1}{2} \biggl( \frac{\sum_{i,j = 1}^{n} (a_{ij})^{2 / ( 1 + \alpha )} ( \delta_{j}(t) - \delta_{i}(t) )^{2}}{ \frac{1}{2}\sum_{i = 1}^{n} \delta_{i}^{2}(t)} V(t) \biggr)^{ ( 1 + \alpha ) / 2}. \end{aligned}$$

Then we get

20 $$\begin{aligned}& \sum_{i,j = 1}^{n} (a_{ij})^{2 / ( 1 + \alpha )} \bigl( \delta_{j}(t) - \delta_{i}(t) \bigr)^{2} = \sum_{i,j = 1}^{n} d_{ij} \bigl( \delta_{j}(t) - \delta_{i}(t) \bigr)^{2} = 2 \boldsymbol{\delta}^{\mathrm{T}}L(D)\boldsymbol{\delta}, \end{aligned}$$

21 $$\begin{aligned}& \frac{2\sum_{i,j = 1}^{n} (a_{ij})^{2 / ( 1 + \alpha )} ( \delta_{j}(t) - \delta_{i}(t) )^{2}}{\sum_{i = 1}^{n} \delta_{i}^{2}(t)} = \frac{4\boldsymbol{\delta}^{\mathrm{T}}L(D)\boldsymbol{\delta}}{ \boldsymbol{\delta}^{\mathrm{T}}\boldsymbol{\delta}} \ge 4\lambda_{2} \bigl( L(D) \bigr) > 0. \end{aligned}$$

Then

22 $$\begin{aligned}& S_{1}(t) \le- \frac{1}{2} \bigl( 4\lambda_{2} \bigl( L(D) \bigr) \bigr)^{ ( 1 + \alpha ) / 2}V^{ ( 1 + \alpha ) / 2}(t), \end{aligned}$$

23 $$\begin{aligned}& S_{2}(t) = \sum_{i = 1}^{n} \delta_{i} (t)e_{i}^{\alpha} (t) \\& \hphantom{S_{2}(t)}= \delta^{\mathrm{T}}(t)e^{\alpha} (t) \le \bigl\Vert \delta(t) \bigr\Vert \bigl\Vert e^{\alpha} (t) \bigr\Vert \\& \hphantom{S_{2}(t)}= \frac{ \Vert \delta(t) \Vert \Vert e(t) \Vert ^{\alpha} V^{ ( 1 + \alpha ) / 2}(t)}{V^{ ( 1 + \alpha ) / 2}(t)} \\& \hphantom{S_{2}(t)}= \frac{ \Vert \delta(t) \Vert \Vert e(t) \Vert ^{\alpha} V^{ ( 1 + \alpha ) / 2}(t)}{2^{ ( 1 + \alpha ) / 2} \Vert \delta \Vert ^{1 + \alpha}} \\& \hphantom{S_{2}(t)}= \frac{ \Vert \delta(t) \Vert ^{ - \alpha} \Vert e(t) \Vert ^{\alpha} V^{ ( 1 + \alpha ) / 2}(t)}{2^{ ( 1 + \alpha ) / 2}}. \end{aligned}$$

Combining the above formulas results in

24 $$\begin{aligned} \frac{dV(t)}{dt} \le&- \frac{ ( 4\lambda_{2} ( D ) )^{ ( 1 + \alpha ) / 2}V^{ ( 1 + \alpha ) / 2}(t)}{2} + \frac{ \Vert \delta(t) \Vert ^{ - \alpha} \Vert e(t) \Vert ^{\alpha} V^{ ( 1 + \alpha ) / 2}(t)}{2^{ ( 1 + \alpha ) / 2}} \\ \le& \biggl( \frac{ \Vert \delta(t) \Vert ^{ - \alpha} \Vert e(t) \Vert ^{\alpha}}{2^{ ( 1 + \alpha ) / 2}} - \frac{ ( 4\lambda_{2} ( D ) )^{ ( 1 + \alpha ) / 2}}{2} \biggr)V^{ ( 1 + \alpha ) / 2}(t). \end{aligned}$$

According to the triggered function of Theorem 1, we get

25 $$ \bigl\Vert e_{i}(t) \bigr\Vert ^{\alpha} \le \frac{\mu ( 4\lambda _{2} ( D ) )^{ ( 1 + \alpha ) / 2} \Vert \delta_{i}(t) \Vert ^{\alpha}}{2^{ ( 1 - \alpha ) / 2}}. $$

Then we get

26 $$ \frac{dV(t)}{dt} \le\frac{1}{2} ( \mu- 1 ) \bigl( 4\lambda_{2} ( D ) \bigr)^{ ( 1 + \alpha ) / 2}V^{ ( 1 + \alpha ) / 2}(t). $$

Therefore, according to Lemma 1, the differential in equation (26) makes $V(t)$ reach zero in finite time. Moreover, the settling time *T* satisfies

27 $$ T \le\frac{4V(0)^{ ( 1 - \alpha ) / 2}}{ ( 1 - \mu ) ( 4\lambda_{2} ( D ) )^{ ( 1 + \alpha ) / 2}(1 - \alpha)}. $$

If $V(t)=0$, then $\boldsymbol{\delta} (t)=0$, which implies that $u _{i} = 0$, $i \in\mathcal{I}_{n}$. Thus, ${x}(t) \in \operatorname{span}(\mathbf{1})$. Therefore, the system stability is guaranteed, and the novel protocol can solve the finite-time average consensus problem. □

#### Remark 1

For each agent, an event is triggered as long as the triggered function satisfies $f_{i} ( t,e_{i}(t),\delta_{i}(t) ) = 0$.

#### Remark 2

The role of the parameter *μ* in the triggered function (8) is adjusting the rate of decrease for the Lyapunov function. From equation (8) we know that when the parameter *μ* is large, the allowable error is large. This means that when *μ* is large, the trigger frequency is low. From equation (27) we know that when *μ* is large, the convergence time is long.

#### Remark 3

Note that if we set $\mu=0$, then the protocol becomes the typical finite-time consensus protocol studied in [19]. However, the finite-time consensus protocol in [19] does not adapt event-triggered control strategy, so that the energy consumption of the system is large.

### Existence of a lower bound for interevent times

In the event-triggered control conditions, the agent cannot exhibit Zeno behavior. Namely, for any initial, the interevent times $\{ \tau _{i + 1} - \tau_{i} \}$ defined by equation (8) are lower bounded by a strictly positive time *τ*. This is proven in the following theorem.

#### Theorem 2

*Consider the multiagent system* (4) *with consensus protocol* (6). *Suppose that the communication topology of the multiagent system is undirected and connected*. *The trigger function is given by equation* (8). *Then the agent cannot exhibit Zeno behavior*. *Moreover*, *for any initial state*, *the interevent times* $\{ \tau_{i + 1} - \tau_{i} \}$ *are lower bounded by* $\tau_{\mathrm{min}}$ *given by*

28 $$ \tau_{\min} = \frac{\mu^{1 / \alpha} \theta}{ \Vert L \Vert ( 1 + \mu^{1 / \alpha} \theta )}, $$

*where* $\theta= ( \frac{ ( 4\lambda_{2} ( D ) )^{ ( 1 + \alpha ) / 2}}{2^{ ( 1 - \alpha ) / 2}} )^{1 / \alpha} $.

#### Proof

Similarly to the main result in [27], define

29 $$ f_{i}(t) = \bigl\Vert e_{i}(t) \bigr\Vert / \bigl\Vert \delta_{i}(t) \bigr\Vert . $$

By differentiating $f _{i}(t)$ we obtain

30 $$\begin{aligned} \frac{d ( \Vert e_{i}(t) \Vert / \Vert \delta_{i}(t) \Vert )}{dt} =& - \frac{e_{i}^{\mathrm{T}}(t)\dot{\delta}_{i}(t)}{ \Vert e_{i}(t) \Vert \Vert \delta_{i}(t) \Vert } - \frac{\delta_{i}^{\mathrm{T}}\dot{\delta}_{i}(t)}{ \Vert \delta_{i}(t) \Vert ^{2}} \frac{ \Vert e_{i}(t) \Vert }{ \Vert \delta_{i}(t) \Vert } \\ \le&\frac{ \Vert e_{i}(t) \Vert }{ \Vert e_{i}(t) \Vert }\frac{ \Vert \dot{\delta}_{i}(t) \Vert }{ \Vert \delta_{i}(t) \Vert } + \frac{ \Vert \dot{\delta}_{i}(t) \Vert }{ \Vert \delta_{i}(t) \Vert }\frac{ \Vert e_{i}(t) \Vert }{ \Vert \delta_{i}(t) \Vert } \\ =& \biggl( 1 + \frac{ \Vert e_{i}(t) \Vert }{ \Vert \delta_{i}(t) \Vert } \biggr)\frac{ \Vert \dot{\delta}_{i}(t) \Vert }{ \Vert \delta_{i}(t) \Vert } \\ \le& \biggl( 1 + \frac{ \Vert e_{i}(t) \Vert }{ \Vert \delta_{i}(t) \Vert } \biggr)\frac{ \Vert L \Vert ( \Vert e_{i}(t) \Vert + \Vert \delta_{i}(t) \Vert )}{ \Vert \delta_{i}(t) \Vert } \\ =& \Vert L \Vert \biggl( 1 + \frac{ \Vert e_{i}(t) \Vert }{ \Vert \delta_{i}(t) \Vert } \biggr)^{2}. \end{aligned}$$

Then

31 $$ \dot{f}_{i}(t) \le \Vert L \Vert \bigl( 1 + f_{i}(t) \bigr)^{2}. $$

Then we can get that the interevent times are bounded by the time as follows:

32 $$ \psi(\tau_{i},0) = \frac{\tau_{i} \Vert L \Vert }{1 - \tau_{i} \Vert L \Vert }. $$

From equation (25) we know

33 $$ \psi(\tau_{i},0) \le \biggl( \frac{\mu ( 4\lambda_{2} ( D ) )^{ ( 1 + \alpha ) / 2}}{2^{ ( 1 - \alpha ) / 2}} \biggr)^{1 / \alpha}. $$

Combining the above formulas results in

34 $$ \tau_{\min} = \frac{\mu^{1 / \alpha} \theta}{ \Vert L \Vert ( 1 + \mu^{1 / \alpha} \theta )}, $$

and the proof is complete. □

#### Remark 4

From equation (34) it is easy to see that the minimum interevent time increases with *μ*.

#### Remark 5

Note that if we set $\alpha=1$ in protocol (6), then the finite-time nonlinear event-triggered control strategy becomes the typical event-triggered linear consensus protocol studied in [9]. However, the event-triggered linear consensus protocol can only make agents achieve consensus asymptotically, whereas the proposed consensus protocol in this paper can solve the consensus problem in finite time.

### Performance analysis

In this subsection, the relationship between convergence time and other factors, including initial state and parameter *α* is studied.

Firstly, we study the relationship between convergence time and initial states. In the consensus problem, rather than the size of the initial states, the disagreement between states is more concerned. By definition, $V(0)$ measures the disagreement of the initial states with final state. From equation (27) we easily see that the convergence time increases as $V(0)$ increases.

Then we study the relationship between convergence time and parameter *α*. Supposing that $T _{u}$ is the upper bound of the communication time by equation (27) we obtain

35 $$ T_{u}(\alpha) = \frac{4V(0)^{ ( 1 - \alpha ) / 2}}{ ( 1 - \mu ) ( 4\lambda_{2} ( D ) )^{ ( 1 + \alpha ) / 2}(1 - \alpha)}. $$

Then

36 $$ \frac{dT_{u}(\alpha)}{d\alpha} = - \frac{2V(0)^{ ( 1 - \alpha ) / 2} ( 4\lambda_{2} ( D ) )^{ ( 1 + \alpha ) / 2}}{ ( 1 - \mu )}\frac{ ( ( 1 - \alpha )\ln V(0) + ( 1 - \alpha )\ln ( 4\lambda_{2} ( D ) ) - 2 )}{ ( ( 4\lambda_{2} ( D ) )^{ ( 1 + \alpha ) / 2}(1 - \alpha) )^{2}}. $$

Letting $dT _{u}( \alpha)/d \alpha=0$, we get $\alpha= 1 - 2 / \ln ( 4V(0)\lambda_{2}(D) )$. Therefore, given that $0< \alpha<1$, if $V(0)\lambda_{2}(D) < E^{2} / 4$, then the convergence time increases as increases; if $V(0)\lambda_{2}(D) > E^{2} / 4$, then the convergence time decreases initially and then increases as *α* becomes large, and when $\alpha= 1 - 2 / \ln ( 4V(0)\lambda_{2}(D) )$, the convergence time gets the minimum value. Generally, the value of $V(0)\lambda_{2}(D)$ is always large, and to reduce the convergence time, we can set $\alpha= 1 - 2 / \ln ( 4V(0)\lambda_{2}(D) )$.

#### Remark 6

Convergence time is defined as the amount of time the system consumes to reach a consensus. The precise convergence time of the studied nonlinear protocol is difficult to obtain. The above conclusions were obtained based on the upper bound of convergence time in equation (27).

## Simulations

**Figure 1:**
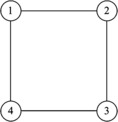
**The communication topology of four agents.**

In this section, the simulations are conducted to verify the efficiency of the conclusions. Consider the multiagent system with $n=4$ agents. The communication topology is shown in Figure 1, and the communication weight is assumed to be 1. Through calculation we can obtain $\lambda_{2}(L) = 2$ and $\Vert L \Vert = 4$. We suppose that the initial state is $[-2, 0, 2, 6]^{\mathrm{T}}$.

**Figure 2:**
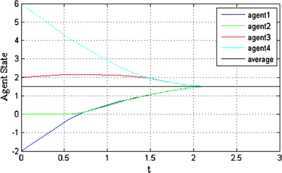
**The trajectories of agents under** $\pmb{\alpha =0.3}$ **,** $\pmb{\mu=0.2}$ **.**

**Figure 3:**
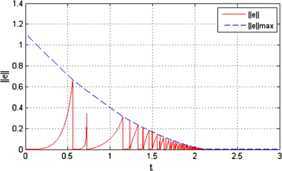
**The evolution of the error norm of agent 1 under** $\pmb{\alpha =0.3}$ **,** $\pmb{\mu=0.2}$ **.**

**Figure 4:**
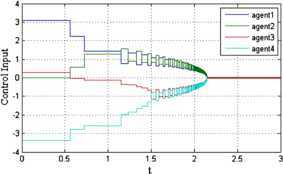
**The evolution of the control input of agents under** $\pmb{\alpha =0.3}$ **,** $\pmb{\mu=0.2}$ **.**

The trajectories of agents under protocol (6) when $\alpha=0.3$ and $\mu =0.2$ are shown in Figure 2. We can see that the final consensus state is $\sum_{i = 1}^{n} x_{i} (0) / 4 = 1.5$. Thus, protocol (6) can solve the finite-time average consensus problem. Figure 3 shows the evolution of the error norm of agent 1 under protocol (6). The red solid line represents the evolution of $\Vert e(t) \Vert $. It stays below the specified state-dependent threshold $\Vert e(t) \Vert _{\max} = ( \frac {\mu ( 4\lambda_{2} ( D ) )^{ ( 1 + \alpha ) / 2}}{2^{ ( 1 - \alpha ) / 2}} )^{1 / \alpha} \Vert \delta (t) \Vert $, which is represented by the blue dotted line in the figure. Figure 4 shows the evolution of the control input of agents under protocol (6). We can see that the control input is a piecewise constant value. Moreover, when the system error norm is small, the control input is small, and when the control input tends to zero, the system reaches equilibrium states.

**Figure 5:**
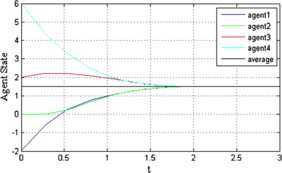
**The trajectories of agents under** $\pmb{\mu=0.2}$ **when** $\pmb{\alpha =0.6}$ **.**

**Figure 6:**
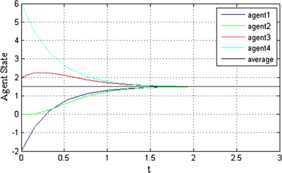
**The trajectories of agents under** $\pmb{\mu=0.2}$ **when** $\pmb{\alpha =0.9}$ **.**

When $\mu=0.2$, the trajectories of agents under $\alpha=0.6$ and $\alpha=0.9$ are shown in Figures 5 and 6, respectively. Comparison with Figure 2 and Figures 5 and 6 shows that if $V(0)\lambda_{2}(D) > E^{2} / 4$ when $\alpha= 1 - 2 / \ln ( 4V(0)\lambda_{2}(D) )$ (approximately 0.6 in this paper), the convergence time is shorter than that when $\alpha=0.3$ and $\alpha=0.9$.

**Figure 7:**
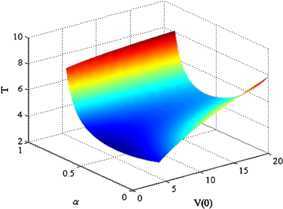
**The relationship between convergence time and parameter** ***α*** **,** $\pmb{V(0)}$ **.**

**Figure 8:**
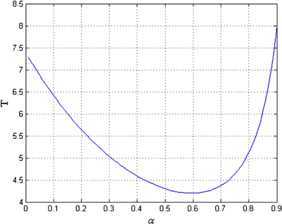
**The relationship between convergence time and parameter** ***α*** **when** $\pmb{V(0)=17.5}$ **.**

Moreover, the relationship between the convergence time and parameter *α*, $V(0)$ is simulated. The result is shown in Figure 7. The relationship between the convergence time and *α* when $V(0) = 17.5$ (the corresponding $V(0)$ for $x(0)$ in this paper) is shown in Figure 8. The two figures show that if $V(0)\lambda_{2}(D) > E^{2} / 4$, then the convergence time decreases initially, then increases as *α* becomes large, and when $\alpha= 1 - 2 / \ln ( 4V(0)\lambda_{2}(D) )$, the convergence time gets the minimum value.

## Conclusions

We presented a novel finite-time average consensus protocol based on event-triggered control strategy for multiagent systems, which guarantees the system stability. The upper bound of convergence time was obtained. The relationship between convergence time and protocol parameter with initial state was analyzed. The following conclusions were obtained from simulations.

1. The proposed protocol can solve the finite-time average consensus problem.
2. The lower bound of the interevent time was obtained to guarantee that there is no Zeno behavior.
3. The larger the difference in the initial state, the longer the convergence time. Moreover, if $V(0)\lambda_{2}(D) > E^{2} / 4$, then the convergence time decreases initially, then increases as *α* becomes large, and when $\alpha= 1 - 2 / \ln ( 4V(0)\lambda_{2}(D) )$, the convergence time gets the minimum value.

In this paper, the authors only considered first-order multiagent systems. Our future works will focus on extending the conclusions to second-order or higher-order multiagent systems with switching topologies, measurement noise, time delays, and so on.
